# Selective Translational Repression of Truncated Proteins from Frameshift Mutation-Derived mRNAs in Tumors 

**DOI:** 10.1371/journal.pbio.0050109

**Published:** 2007-04-24

**Authors:** Kwon Tae You, Long Shan Li, Nam-Gyun Kim, Hyun Ju Kang, Kwi Hye Koh, Yong-Joon Chwae, Kyoung Mi Kim, Yoon Ki Kim, Sung Mi Park, Sung Key Jang, Hoguen Kim

**Affiliations:** 1 Department of Pathology, Yonsei University College of Medicine, Seoul, Korea; 2 Brain Korea 21 Projects for Medical Sciences, Yonsei University College of Medicine, Seoul, Korea; 3 Department of Microbiology, Pochon Cha University College of Medicine, Pocheon, Gyeonggi, Korea; 4 School of Life Sciences and Biotechnology, Korea University, Seoul, Korea; 5 Department of Life Science, Pohang University of Science and Technology, Pohang, Kyungbuk, Korea; University of Wisconsin, United States of America

## Abstract

Frameshift and nonsense mutations are common in tumors with microsatellite instability, and mRNAs from these mutated genes have premature termination codons (PTCs). Abnormal mRNAs containing PTCs are normally degraded by the nonsense-mediated mRNA decay (NMD) system. However, PTCs located within 50–55 nucleotides of the last exon–exon junction are not recognized by NMD (NMD-irrelevant), and some PTC-containing mRNAs can escape from the NMD system (NMD-escape). We investigated protein expression from NMD-irrelevant and NMD-escape PTC-containing mRNAs by Western blotting and transfection assays. We demonstrated that transfection of NMD-irrelevant PTC-containing genomic DNA of *MARCKS* generates truncated protein. In contrast, NMD-escape PTC-containing versions of *hMSH3* and *TGFBR2* generate normal levels of mRNA, but do not generate detectable levels of protein. Transfection of NMD-escape mutant *TGFBR2* genomic DNA failed to generate expression of truncated proteins, whereas transfection of wild-type *TGFBR2* genomic DNA or mutant PTC-containing *TGFBR2* cDNA generated expression of wild-type protein and truncated protein, respectively. Our findings suggest a novel mechanism of gene expression regulation for PTC-containing mRNAs in which the deleterious transcripts are regulated either by NMD or translational repression.

## Author Summary

A class of mutations found in many cancers introduces aberrant termination signals during the synthesis of mRNA. In mammalian cells, abnormal mRNAs containing premature termination codons (PTCs) are normally degraded by a process called nonsense-mediated mRNA decay (NMD), thus avoiding potentially deleterious effects from abnormal protein production. However, some PTC-containing mRNAs are known to escape from NMD. By screening protein expression from genes with serious mutations in colon cancers, we confirmed that PTC-containing mRNAs of some genes escape from NMD. However, their abnormal proteins were not found in the tumor cells. To study the means by which these proteins were regulated, we transfected separate cell lines with NMD-escape mutant genomic DNA, wild-type genomic DNA, and mutant cDNA. We found that truncated proteins are not generated from the NMD-escape mutant genomic DNA, whereas wild-type protein and truncated protein were generated normally. These results indicate that the translation of PTC-containing mutant mRNAs is repressed in the cytoplasm.

## Abstract 3

A novel mechanism for regulating the products of nonsense mRNAs that contain premature termination codons involves repression at the level of translation.

## Introduction

A subset of colorectal carcinomas exhibit a molecular phenotype commonly referred to as high microsatellite instability (MSI-H) [1]. The microsatellite instability (MSI) pathway begins with the inactivation of one of a group of genes responsible for DNA nucleotide mismatch repair (MMR), which leads to extensive mutations in both repetitive and non-repetitive DNA sequences [2–4]. The mechanism of tumorigenesis in MSI-H tumors is thought to involve frameshift mutations of microsatellite repeats within the coding regions of affected genes, and the inactivation of these genes is believed to contribute directly to tumor development and progression [5,6]. The frameshift mutations observed in the affected genes are expected to generate previously undescribed amino acid sequences in the C-terminal part of the respective proteins (Figure S1). If abnormal mRNAs and proteins are generated from the frameshift-mutated genes, tumor-specific antigen may be generated. High peritumoral lymphocytic infiltration and a relatively good prognosis have been reported in MSI-H tumors [7,8].

One of the important consequences of frameshift mutations is the formation of premature termination codons (PTCs). In mammalian cells, mRNAs containing a PTC due to a nonsense mutation or a frameshift mutation are recognized and degraded by nonsense-mediated mRNA decay (NMD), thus eliminating the production of the potentially deleterious truncated proteins [9,10]. NMD of mRNAs carrying PTCs is mediated through the recognition of the PTC by its position relative to the 3′-most last exon–exon junction. As a general rule, mammalian transcripts that contain a PTC more than 50–55 nucleotides (nt) upstream of the last exon–exon junction will be subjected to NMD [11,12].

Although PTC formation in frameshift mutation-derived mRNAs and their subsequent degradation through NMD is widely accepted, PTCs located within 50–55 nt or downstream of the last exon–exon junction are not recognized by NMD (NMD-irrelevant), and some mRNAs with PTCs more than 50–55 nt upstream of their last exon–exon junction are not degraded by NMD (NMD-escape) [13,14]. In MSI-H tumors, several NMD-sensitive or NMD-escape PTC-containing mRNAs have been reported. A previous study compared the total mRNAs of affected genes from various cell lines [15]. However, this study did not differentiate the proportion of wild-type and mutant mRNAs, and did not confirm the mutant mRNAs through sequencing. This study also did not consider that the amount of mRNA from the affected genes might vary between cell lines. Moreover, the expression statuses and biological effects of the NMD-escape PTC-containing mRNAs are essentially unknown.

In order to clarify the protein expression status of affected genes with frameshift mutations and the role of NMD in these mutated genes, we selected MSI-H cancers as a model system because these cancers have accumulated genes with frameshift mutations, and the mRNAs expected from these mutated genes contain PTCs. We analyzed the expression of 20 mutant mRNAs from 12 genes and evaluated their regulation along with the regulation of associated proteins. We demonstrate that some PTC-containing mRNAs escaped from NMD, but did not generate truncated proteins, indicating that PTC-containing transcripts can be regulated either by NMD or translational repression.

## Results

### Genes with Frameshift Mutations Form PTCs in Their mRNAs

To examine the effect of NMD on the affected genes with frameshift mutations in MSI-H tumors, we selected 12 genes from MSI-H tumors based on the reported frameshift mutation frequencies greater than 30% *(ABCF1, ACVR2, hMSH3, hMSH6, hRad50, MARCKS, PRKWNK1, RFC3, SEC63, TAF1B, TCF-4,* and *TGFBR2).* We used genome sequencing of these 12 genes to identify frameshift mutation status. In these 12 genes, we identified 20 frameshift mutations that fell into three categories: 12 mutations were single base pair (bp) deletions, six were 2-bp deletions, and two were single bp insertions in coding mononucleotide repeats (cMNR) (Table S1). All 20 frameshift mutations of the 12 genes resulted in mRNAs containing a PTC (Table 1).

**Table 1:**
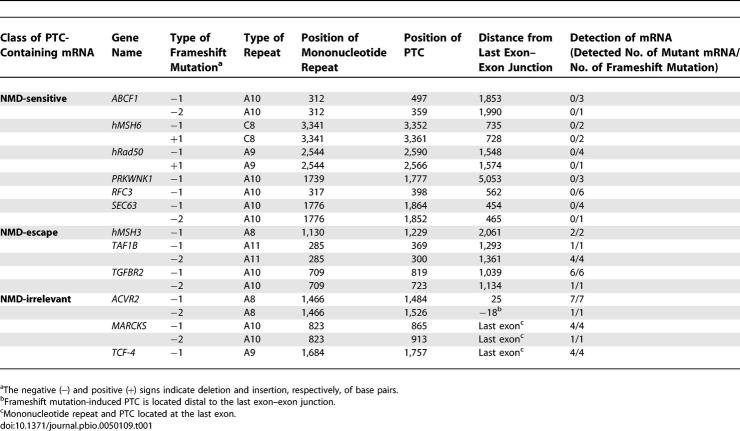
Expressions of Frameshift Mutant mRNAs in MMR-Deficient Colorectal Cancer Cell Lines

### Identification of NMD-Escape PTC-Containing mRNAs

We analyzed mRNA expression of the 12 genes by reverse transcriptase PCR (RT-PCR) in seven MMR-deficient (LS174T, HCT-8, SNU C2A, SNU C4, DLD-1, HCT116, and LOVO) and three MMR-proficient (NCI-H508, SW480, and HT29) colorectal cancer cell lines. Primers were designed to contain at least one exon–exon junction region and to amplify the coding repeat sequences (Table S2). Of the 20 frameshift mutations in the genomic DNA, mutation-derived transcripts were detected from ten alleles representing six genes *(hMSH3, TAF1B, TGFBR2, ACVR2, MARCKS,* and *TCF-4),* whereas ten alleles representing six genes *(ABCF1, hMSH6, hRad50, PRKWNK1, RFC3,* and *SEC63)* did not generate frameshift mutation-derived transcripts. No differences in expression of frameshift mutation-derived mRNA were observed between cell lines. Of the ten transcripts with frameshift mutations, five transcripts (representing three genes: *hMSH3, TAF1B,* and *TGFBR2*) had PTCs more than 50–55 nt upstream of the last exon–exon junction and were therefore expected to be degraded by NMD but instead escaped from NMD (NMD-escape). On the other hand, the five remaining transcripts (representing three genes: *ACVR2, MARCKS,* and *TCF-4*) had PTCs within 50–55 nt upstream of the last exon–exon junction and were therefore expected to be irrelevant to NMD (NMD-irrelevant). Accordingly, the 20 transcripts from 12 genes were classified as NMD-sensitive, NMD-escape, and NMD-irrelevant (Table 1).

**Figure 1:**
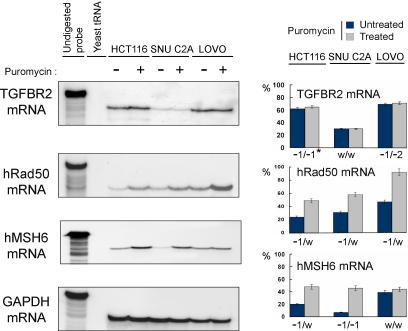
Measurement of Degraded Premature Termination Codon-Containing mRNAs by RPA Analysis No loss of *TGFBR2* mutant transcripts was noted after puromycin treatment. In contrast, the amount of *hRad50* and *hMSH6* mRNA doubled after puromycin treatment. *GAPDH* was used for standardization. The asterisk indicates mutation status of each gene: (−1) denotes a 1-bp deletion in the cMNR, (w) denotes no mutation in the cMNR, (+1) denotes a 1-bp insertion in the cMNR, and (−2) denotes a 2-bp deletion in the cMNR.

In order to confirm the effect of NMD on the NMD-sensitive and NMD-escape PTC-containing mRNAs, we used RT-PCR and a ribonuclease protection assay (RPA) to analyze the expression of the target gene mRNAs after treatment with puromycin, a translation inhibitor. In the five NMD-escape alleles that generated detectable frameshift mutation-derived mRNAs, no expression differences were found after puromycin treatment. In the ten NMD-sensitive alleles that produced no detectable frameshift mutation-derived mRNAs, mutant transcripts were detected after puromycin treatment (Figure S2). We analyzed the amount of two degraded NMD-sensitive transcripts, hRad50 and hMSH6, by RPA and found a total loss of mutant transcripts, as evidenced by a 2-fold increase in hRad50 and hMSH6 products after puromycin treatment. In contrast, there was no loss of TGFBR2 mutant mRNA, an NMD-escape transcript, because the amount of product was unchanged after puromycin treatment (Figure 1).

Next, we evaluated the effect of down-regulating UPF1 or UPF2, which are key NMD factors, on the stability of the frameshift mutation-derived mRNAs, hRad50 and hMSH6, using specific small interfering RNA (siRNA). Upon the treatment of luciferase siRNA, expression of the mutation-derived hRad50 and hMSH6 mRNAs were not detected in the cell lines with hRad50 and hMSH6 mutations. In contrast, down-regulating UPF1 or UPF2 abundantly increased the frameshift mutation-derived mRNAs, as confirmed by RT-PCR, and sequence analysis. These findings indicate that frameshift mutation-derived mRNAs of hRad50 and hMSH6 are recognized and degraded by the NMD system (Figure S3).

### Truncated Proteins from NMD-Escape PTC-Containing mRNAs of hMSH3 Are Not Detected by Western Blotting

**Figure 2:**
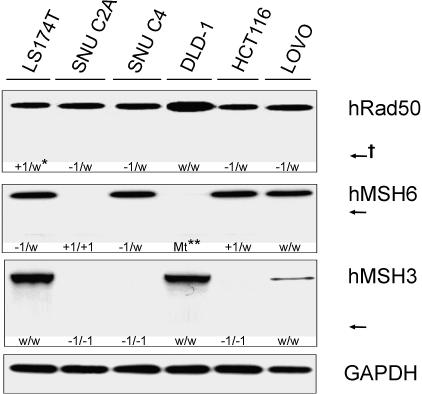
Western Blotting Analysis of NMD-Sensitive and NMD-Escape Proteins Truncated proteins were not detected in NMD-sensitive *(hRad50* and *hMSH6)* genes, whereas wild-type proteins were detected in the cell lines containing the wild-type allele. In the NMD-escape *hMSH3* gene, wild-type hMSH3 proteins were detected in the cell lines containing the wild-type allele. However, no truncated proteins of hMSH3 were detected in the cell lines with frameshift mutation. GAPDH was used as a loading control. The single asterisk indicates mutation status of each gene: (−1) denotes a 1-bp deletion in the cMNR, (w) denotes no mutation in the cMNR, and (+1) denotes 1-bp insertion in the cMNR. The double asterisk indicates frameshift mutations of the other coding regions present in both alleles of DLD-1 [38]. The dagger indicates that the expected size of the truncated protein is indicated by the arrow.

In order to determine if the truncated protein products from PTC-containing mRNAs can be detected, we first performed Western blotting analyses using antibodies directed against the N-terminus of hRad50, hMSH6, and hMSH3. Truncated proteins were not detected for the NMD-sensitive *(hRad50* and *hMSH6)* genes, whereas wild-type proteins were detected in the cell lines containing the wild-type allele. These results support the previous finding that NMD-sensitive PTC-containing mRNAs are degraded by the NMD system. In the NMD-escape *hMSH3* gene, we detected full-length hMSH3 proteins in cell lines with no mutations or with monoallelic mutations in these genes; however, we could not detect truncated hMSH3 proteins in any of the cell lines carrying frameshift mutations (Figure 2). The hMSH3 antibody detected the truncated proteins from the cell lines transfected with mutant hMSH3 cDNA, indicating that the antibodies used in our experiments specifically react with the N-terminal region of hMSH3 protein (Figure S4). Furthermore, we could not detect the truncated proteins of *hMSH3* genes after treatment with the proteasome inhibitors MG132 or E64 (unpublished data), which excludes the possibility of rapid degradation of mutated proteins.

### Selective Translational Repression of Mutant TGFBR2 following Normal Splicing

We interpreted the failure to detect truncated protein from the NMD-escape PTC-containing mRNAs as follows: (1) truncated proteins were generated, but at an amount not sufficient for detection by Western blotting, (2) truncated proteins were generated, but then rapidly degraded, or (3) truncated proteins were not generated from the mutant mRNA.

**Figure 3:**
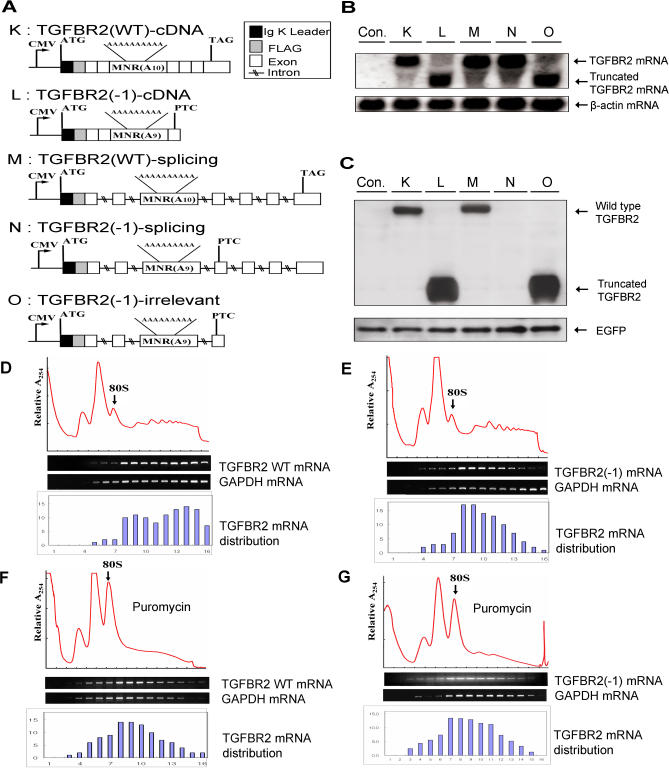
Transfection Assay of the TGFBR2 Vector (A) Schematic diagram of construct K (TGFBR2 wild-type cDNA), construct L (TGFBR2(−1)-deleted cDNA), construct M (TGFBR2 wild-type genomic DNA), construct N (TGFBR2(−1)-deleted genomic DNA), and construct O (TGFBR2(−1)-deleted genomic DNA with a PTC artificially located in the last exon). (B) Analysis of TGFBR2 mRNA by Northern blotting. The abundant expression of TGFBR2 mRNAs from the transfected constructs is shown. β-Actin was used as a RNA loading control. Con. denotes the control vector pSecTag2B. (C) Western blotting using anti-FLAG. Protein expression of transfected TGFBR2 constructs. Cell lines transfected with the constructs K and M expressed wild-type TGFBR2. Cell lines transfected with the constructs L and O expressed truncated TGFBR2. However, cell lines transfected with construct N did not express truncated TGFBR2, indicating translational repression after normal splicing. Con. denotes the control vector pSecTag2B. Enhanced green fluorescent protein (EGFP) was used as a transfection control. (D) Sucrose gradient fractionation of cytoplasmic extracts from cells expressing TGFBR2(WT)-splicing mRNA. (E) Sucrose gradient fractionation of cytoplasmic extracts from cells expressing TGFBR2(−1)-splicing mRNA. (F) TGFBR2(WT)-splicing transfected cells were treated with puromycin prior to lysis and fractionation. (G) TGFBR2(−1)-splicing transfected cells were treated with puromycin prior to lysis and fractionation. In (D, E, F, and G), RNA extracted from each fraction was subjected to RT-PCR. Endogenous GAPDH mRNA was used as a control. TGFBR2(WT) mRNA was found in the polysome-containing fractions similar to endogenous GAPDH mRNA. However, a greater percentage of PTC-containing TGFBR2(−1) mRNA was found in the fractions containing ribosomal subunits and monosomes. The plots denote quantitative representation of TGFBR2 mRNA distribution in polysome gradients. Relative mRNA levels in each fraction were calculated as a percentage of the total. Results from two independently performed experiments did not vary, and the plot represents the average of the two independent experiments.

To rule out an insufficient amount of endogenous truncated proteins, we constructed expression plasmids with NMD-escape PTC-containing genomic DNA or cDNA of *TGFBR2:* (1) wild-type cDNA of *TGFBR2* (K: TGFBR2(WT)-cDNA), (2) PTC-containing mutant cDNA of *TGFBR2* without downstream exons and introns (L: TGFBR2(−1)-cDNA), (3) wild-type *TGFBR2* genomic DNA (M: TGFBR2(WT)-splicing), (4) mutant *TGFBR2* genomic DNA with a 1-bp deletion (N: TGFBR2(−1)-splicing), and (5) mutant *TGFBR2* genomic DNA with a 1-bp deletion and a PTC artificially located in the last exon (O: TGFBR2(−1)-irrelevant) (Figure 3A). Among three NMD-escape PTC-containing mutated genes that we found in MSI-H tumors, we selected *TGFBR2*. *TAF1B* and *hMSH3* were excluded because of their large size and number of exons, which result in the failure or inefficient transfection of genomic DNA.

In all of the constructs described above, the nucleotide sequences encoding FLAG peptide was introduced immediately downstream of the initiation codon, which allows for detection of the encoded proteins by Western blotting. These vectors were designed to differentiate the effect of spliced wild-type mRNA, spliced mutant NMD-escape mRNA, and spliced mutant NMD-irrelevant mRNA in terms of truncated protein expression. We observed abundant expression of PTC-containing *TGFBR2* mRNA in cell lines transfected with TGFBR2(−1)-cDNA, TGFBR2(−1)-splicing, and TGFBR2(−1)-irrelevant (Figure 3B). Cell lines transfected with TGFBR2(WT)-splicing, TGFBR2(−1)-splicing, and TGFBR2(−1)-irrelevant showed accurate splicing, and all normal and mutant mRNA products were confirmed by sequence analysis (unpublished data)**.** A semi-quantitative RT-PCR analysis designed to detect exogenous *TGFBR2* mRNA showed similar and abundant amounts of *TGFBR2* mRNA expression in all of the cell lines transfected with the five different constructs (unpublished data). In this analysis of protein expression using the anti-FLAG antibody, we demonstrated the expression of wild-type TGFBR2 protein in cell lines transfected with TGFBR2(WT)-cDNA and TGFBR2(WT)-splicing. We also demonstrated the expression of truncated TGFBR2 protein in cell lines transfected with TGFBR2(−1)-cDNA and TGFBR2(−1)-irrelevant. Intriguingly, we could not detect any TGFBR2 protein in cell lines transfected with TGFBR2(−1)-splicing, indicating a selective translational repression of NMD-escape mutant mRNA (Figure 3C).

### PTC-Containing TGFBR2 mRNA Does Not Associate with Polysomes

In order to confirm that translational repression is responsible for the failure to detect truncated protein from PTC-containing TGFBR2 mRNA, we examined the mRNA distribution of TGFBR2(WT)-splicing and TGFBR2(−1)-splicing by polysome analysis.

In the cell line with the TGFBR2(WT)-splicing vector, TGFBR2(WT)-splicing mRNA was found in the polysome-containing fractions similar to endogenous GAPDH mRNA (Figure 3D). However, in the cell line with the TGFBR2(−1)-splicing vector, a greater percentage of TGFBR2(−1)-splicing mRNA was found in the fractions that contained ribosomal subunits and monosomes, whereas endogenous GAPDH mRNA co-sedimented with polysomes (Figure 3E). Furthermore, upon the treatment of puromycin, a greater percentage of TGFBR2(WT)-splicing mRNA and endogenous GAPDH mRNA were shifted into ribosomal subunits and monosome-containing fractions (Figure 3F). In order to rule out the possibility that the weak polysome association of TGFBR2(−1)-splicing mRNA is due to its shorter open reading frame as compared to the TGFBR2(WT)-splicing mRNA, we repeated the same experiment using the TGFBR2(−1)-splicing vector with puromycin treatment. The results show that there is no significant difference in the cell line transfected with TGFBR2(−1)-splicing after puromycin treatment (Figure 3E and 3G). These results indicate that (1) the sedimentation of TGFBR2(WT)-splicing mRNA in heavy fractions was due to polysome association, and (2) the shift of TGFBR2(−1)-splicing mRNA into ribosomal subunits and monosome-containing fractions is due to translational repression, similar to TGFBR2(WT)-splicing mRNA and TGFBR2(−1)-splicing mRNA treated with puromycin (Figure 3E–3G). This novel mechanism, whereby PTC recognition itself triggers translational repression, is referred to as nonsense-mediated translational repression (NMTR).

### Factors Involved in Translational Repression of NMD-Escape Mutant mRNA

We demonstrated the selective translational repression of the NMD-escape mutant TGFBR2(−1)-splicing mRNA after normal splicing, and this repression was not found in the NMD-irrelevant mutant TGFBR2 mRNA, which lacks a downstream sequence of the termination codon. Therefore, we examined other possible factors influencing the expression of the truncated protein by: (1) changing the 3′ UTR length (the length between the PTC and poly(A) tail) to check the possible effect of 3′ UTR length on translational repression [16], (2) treating with a proteasome inhibitor (MG132) in the cell lines transfected with TGFBR2(−1)-splicing and TGFBR2(−1)-irrelevant to rule out that the truncated proteins are generated but rapidly degraded by the proteasome, and (3) down-regulating key NMD factors, UPF1 and UPF2, to evaluate whether NMD factors are involved in the translational repression of NMD-escape PTC-containing spliced TGFBR2 mutant mRNA.

**Figure 4:**
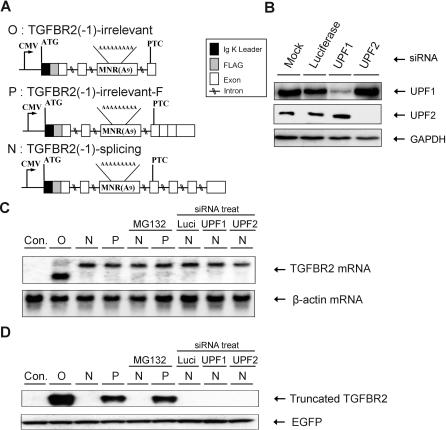
Both 3′ UTR and Splicing Are Important for Translational Suppression of PTC-Containing TGFBR2 mRNA (A) Schematic diagram of constructs P (NMD-irrelevant-type TGFBR2(−1)-deleted construct containing a full coding sequence), O, and N. (B) Western blotting using anti-UPF1, anti-UPF2, or as a loading control, anti-GAPDH. (C) Analysis of TGFBR2 mRNA by Northern blotting. The abundant expression of TGFBR2 mRNAs from the transfected constructs is shown. β-Actin was used as a RNA loading control. (D) Protein expression of transfected TGFBR2 constructs. Truncated proteins are not expressed in the cell lines transfected with TGFBR2(−1)-splicing, whereas, truncated proteins are expressed by cell lines transfected with TGFBR2(−1)-irrelevant and TGFBR2(−1)-irrelevant-F. The amount of truncated protein from the TGFBR2(−1)-irrelevant-F is approximately 35% of TGFBR2(−1)-irrelevant, indicating that the 3′ UTR length or *cis*-element(s) affect translational efficiency. Truncated proteins are not detected from cell lines transfected with TGFBR2(−1)-splicing when treated with MG132, a proteasome inhibitor. Truncated proteins are not generated from cell lines transfected with TGFBR2(−1)-splicing when UPF1 and UPF2 are knocked down by siRNA, indicating that UPF1 and UPF2 do not have an important role in the translational repression of TGFBR2(−1)-splicing mRNA. Enhanced green fluorescent protein (EGFP) was used for transfection control. Con. denotes the control vector pSecTag2B. Luci denotes luciferase siRNA.

In order to change the 3′ UTR length, we constructed another TGFBR2(−1)-irrelevant with a full-length cDNA sequence spanning from the PTC to the 3′ end of *TGFBR2* (P: TGFBR2(−1)-irrelevant-F) (Figure 4A). When the genomic DNAs of TGFBR2(−1)-splicing, TGFBR2(−1)-irrelevant, and TGFBR2(−1)-irrelevant-F were transfected, normal splicing and a large amount of mutant mRNAs were present in all three cell lines transfected with the different genomic DNAs (Figure 4C). However, no proteins were expressed in the cell lines transfected with TGFBR2(−1)-splicing, whereas a large amount of truncated proteins were expressed in the cell lines transfected with TGFBR2(−1)-irrelevant. In the cell lines transfected with TGFBR2(−1)-irrelevant-F, the amount of truncated proteins was reduced to about 35% of that of the cell lines transfected with TGFBR2(−1)-irrelevant. These findings indicate that the 3′ UTR length itself or specific *cis*-acting element(s) within the 3′ UTR seem to contribute to the translational inhibition of TGFBR2(−1) mRNA. However, more importantly, a splicing event downstream of the PTC may be involved in NMTR because truncated proteins are expressed in cells transfected with TGFBR2(−1)-irrelevant-F, but not in cells transfected with TGFBR2(−1)-splicing (Figure 4D).

We excluded the possibility that mutated proteins are generated, but then rapidly degraded by the proteasome, because cell lines transfected with TGFBR2(−1)-splicing and treated with MG132, a proteasome inhibitor, demonstrated no truncated proteins. In contrast, the cell lines transfected with TGFBR2(−1)-irrelevant-F and treated with MG132 demonstrated similar amounts of truncated proteins compared to cell lines only transfected with TGFBR2(−1)-irrelevant-F (Figure 4D).

Finally, we evaluated whether key NMD factors are involved in NMTR. We expected that the most significant difference between PTC-containing TGFBR2 mRNA and PTC-containing TGFBR2 mRNA, which lacks an intron downstream of the PTC, would be the presence of exon junction complexes (EJCs) behind the PTC. An EJC recruits the NMD factors, UPF1 and UPF2, which play a key role in mRNA quality control. We treated cells with UPF1 and UPF2 siRNA in order to elucidate whether these two NMD factors are involved in NMTR. The level of UPF1 was down-regulated to about 20% of normal, where normal is defined as the level in the presence of the nonspecific control, luciferase siRNA, whereas the level of UPF2 was down-regulated to about 10% of normal (Figure 4B). Treatment of any of the siRNAs failed to produce truncated proteins in the cell lines transfected with TGFBR2(−1)-splicing, indicating that at least these two NMD factors do not play an important role in the NMTR of NMD-escape mutant TGFBR2 mRNA (Figure 4D).

### NMD-Irrelevant Mutant mRNA of MARCKS Generates Truncated Proteins

**Figure 5:**
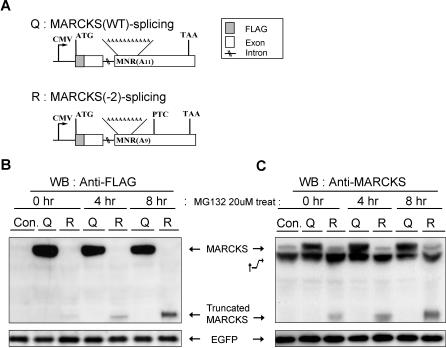
Truncated Protein Expression from Mutant NMD-Irrelevant *MARCKS* mRNA (A) Schematic diagram of construct Q (MARCKS wild-type genomic DNA) and construct R (MARCKS(−2) deleted genomic DNA). (B) Western blotting (WB) analysis of total protein (30 μg) isolated from transiently transfected cell lines with constructs Q or R. An antibody against FLAG was used as the primary antibody. (C) Western blotting (WB) analysis of total protein (30 μg) isolated from transiently transfected cell lines with the constructs Q or R was performed using an antibody against MARCKS. Enhanced green fluorescent protein (EGFP) was used as a control for transfection efficiency. Comparable results were obtained in at least two independent experiments. Asterisks (*) denote endogenous MARCKS reacted with anti-MARCKS antibody; Con. denotes the control vector pcDNA3.1(+). The dagger indicates uncharacterized protein that did not interfere with experimental interpretations.

We then examined whether NMD-irrelevant PTC-containing mRNA can generate truncated protein. We selected one NMD-irrelevant mRNA, mutant *MARCKS,* and performed a transfection assay using wild-type *MARCKS* genomic DNA (MARCKS(WT)-splicing) and mutant *MARCKS* genomic DNA with a 2-bp deletion (MARCKS(−2)-splicing) (Figure 5A).

We found expression of wild-type and truncated protein in cell lines transfected with MARCKS(WT)-splicing and MARCKS(−2)-splicing, respectively, by Western blotting with an anti-FLAG antibody (Figure 5B). To verify that these protein products were identical to MARCKS, we performed Western blotting with the anti-MARCKS antibody and confirmed the expression of wild-type and truncated proteins (Figure 5C). We also found that the truncated MARCKS protein is subject to active proteasome-mediated degradation; the amount of truncated MARCKS protein increased with time when cells were treated with MG132, a proteasome inhibitor (Figure 5B and 5C).

## Discussion

**Figure 6:**
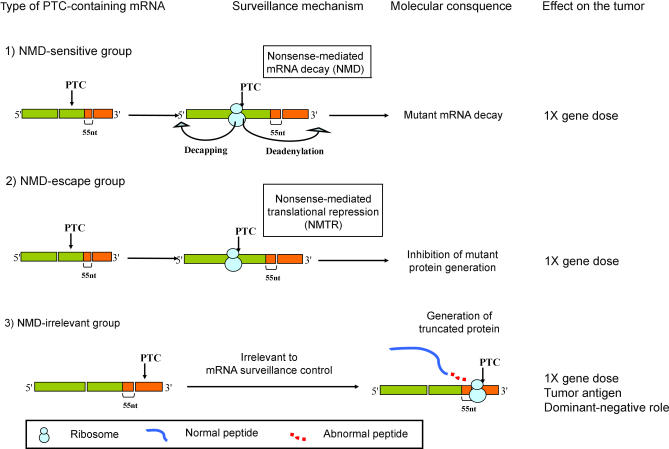
Schematic Model for the Functional Consequences of the Three Classes of PTC-Containing mRNAs NMD-sensitive mRNAs are degraded by the NMD system. NMD-escape mRNAs are not degraded by the NMD system, but experience repression of protein expression. NMD-irrelevant mRNAs are not recognized by the NMD system and generate truncated proteins.

In this study, we found that some PTC-containing mRNAs are not degraded by the NMD system, and their protein translations are repressed. We therefore suggest that PTC-containing mRNAs resulting from frameshift mutations can be classified into three groups: NMD-sensitive mRNAs, which are degraded by the NMD system; NMD-escape mRNAs, which are not degraded by the NMD system, but do experience repression of protein expression; and NMD-irrelevant mRNAs, which are not recognized by the NMD system, and generate truncated proteins. Our findings indicate that both NMD and NMTR, an additional surveillance mechanism for translational control, are involved in the recognition of PTC and suppression of truncated protein from PTC-containing genes that can be deleterious to cell function (Figure 6).

NMD is a quality control-based surveillance mechanism that protects cells from the potentially dominant negative effects of truncated mutant proteins. The primary role of the NMD pathway is to eliminate nonsense transcripts that result from faulty transcription, alternative splicing, or somatic mutation [17,18]. This pathway selectively degrades mRNAs that prematurely terminate translation due to a frameshift or nonsense mutation. Although NMD is a quality control-based surveillance mechanism, avoidance of NMD by PTC-containing mRNAs has been reported for the mutated genes of many diseases. Moreover, about one third of the alternative transcripts in cells are expected to contain PTCs due to splicing errors and regulated unproductive splicing and translation (RUST). Some of these PTC-containing mRNAs belong to the NMD-escape variety [14,15]. If translated, these NMD-escape mRNAs could produce truncated proteins that may critically interfere with cell viability. Among the PTC-containing mRNAs, some PTCs, which are called NMD-irrelevant mRNAs, are located within 50–55 nt or downstream of the last exon–exon junction and are not detected by NMD. Proteins generated from these types of PTC-containing mRNAs and their causal relationship to specific diseases have been well documented [19–21]. However, there are no reports describing translated proteins from mutation-derived NMD-escape mRNAs, although many NMD-escape PTC-containing mRNAs have been reported [22–24].

In this study, we demonstrated that NMD-escape TGFBR2 mRNA is subject to NMTR. Our transfection study of *TGFBR2* constructs demonstrated that PTC-containing mRNAs from mutant *TGFBR2* were abundant after transfection of mutant cDNA and mutant *TGFBR2* genomic DNAs with a 1-bp deletion. However, truncated proteins were only detected in the cell lines transfected with mutant TGFBR2 cDNA, and no truncated proteins were detected in the cell lines transfected with mutant *TGFBR2* genomic DNAs with a 1-bp deletion. In contrast, strong expression of TGFBR2 protein was observed in the cell lines transfected with wild-type *TGFBR2* genomic DNA. Next, we confirmed using polysome analysis that the lack of truncated protein translated from PTC-containing TGFBR2 mRNA is due to translational repression, not instability of TGFBR2 mRNA.

The major expected differences between the two PTC-containing TGFBR2 mutant mRNAs and mRNA from the cDNA of *TGFBR2* or *TGFBR2* genomic DNA are the deposition of the EJC and the possible recruitment of NMD factors to the mRNA during translation termination. We also confirmed the NMTR by demonstrating that mutant mRNA and truncated proteins were efficiently expressed in the cell line transfected with mutant *TGFBR2* genomic DNA containing a PTC in the last exon without downstream introns (Figures 3C and 4D). We therefore suspected that EJC proteins and/or NMD factors might play an important role in the NMTR. It is well known that NMD recognizes PTC and downstream splicing events that deposit an EJC at an exon–exon junction. The EJC is composed of proteins involved in splicing and the subsequent steps of mRNA transport and translation. EIF4A3, RNPS1, Y14, and MAGOH are involved in EJC formation, and the EJC–mRNA complex is then exported to the cytoplasm together with nuclear cap binding proteins CBP80/20 and nuclear poly(A) binding protein 2 (PABP2) [25–28]. The mRNA then recruits UPF2 and undergoes a so-called “pioneer” round of translation during mRNA export. NMD occurs when translation terminates more than 50–55 nt upstream of the last exon–exon junction. Transient SURF formation at the termination codon, which is composed of Smg1, UPF1, and translation termination factors eRF1–eRF3, is thought to interact with the downstream EJC so as to trigger phosphorylation of UPF1 and thereby elicit NMD [26–30]. In this study, we demonstrated that key NMD factors, UPF1 and UPF2, did not play an important role in the NMTR, which is evidenced by the fact that treatment of UPF1 and UPF2 siRNA did not produce truncated proteins in cell lines transfected with TGFBR2(−1)-splicing, even though both siRNAs drastically down-regulate endogenous UPF1 and UPF2. Moreover, down-regulating Y14 or EIF4A3, which are EJC components, using siRNA failed to restore translational repression of TGFBR2(−1)-splicing (unpublished data). Our results indicate that NMTR is at work on the NMD-escape PTC-containing TGFBR2 mRNA by some unknown surveillance mechanism. The involvement of another messenger ribonucleoprotein particle (mRNP) complex in this translational repression is essentially unknown. Future studies should be focused on the role of translational repression of the various RNA binding proteins in the PTC-containing mRNP complex.

Several recent reports have demonstrated the importance of termination codon context, especially 3′ UTR length, in PTC recognition [16]. Therefore, we tested two TGFBR2(−1)-irrelevant vectors with short and extended 3′ UTR lengths. If the PTC-containing, 3′ UTR-extended construct failed to produce truncated protein, then unlike NMD, splicing and EJCs may not be involved in the mechanism of NMTR. In this experiment, we demonstrated a 65% reduction of truncated protein in the cell lines transfected with TGFBR2(−1)-irrelevant vector with an extended 3′ UTR length. These findings indicate that 3′ UTR length is an important factor; however, other important factors are involved in NMTR. Because NMTR depends on 3′ UTR length or a putative *cis*-element residing in the 3′ UTR, it is, in part, reminiscent of EJC-independent NMD. A PTC within the penultimate exon of the β-globin or TPI gene elicits NMD depending on the position of PTC relative to the last EJC [12,31]. However, for a PTC within the penultimate exon that normally elicits NMD, deleting the last intron fails to eliminate NMD, indicating that the last exon has a so-called “failsafe” sequence that allows for PTC recognition and triggers NMD in the absence of a downstream EJC. Intriguingly, this element requires that splicing occur upstream of the PTC because the PTC-containing mRNA that is derived from an intronless TPI or β-globin gene is immune to NMD [12]. It remains to be clarified whether NMTR also requires a splicing event upstream of the PTC. Recently, another case for EJC-independent NMD has been reported in immunoglobulin-μ mRNA [16]. Similar to NMTR, EJC-independent NMD of this mRNA depends on the 3′ UTR length. However, the mode of PTC recognition looks quite different between NMTR and EJC-independent NMD of this mRNA, in the sense that NMTR does not require the NMD factors, UPF1 and UPF2, as shown in our study. Important questions of whether mRNAs targeted by EJC-independent NMD are subject to NMTR should be addressed.

Marked degradation of PTC-containing mRNAs and decreased protein synthesis from PTC-containing mRNAs by the NMD system have been reported in yeast [32]. Another important mRNA surveillance mechanism, nonsense-mediated altered splicing (NAS), has been reported in mammalian cells [33]. NAS induces alternative splicing in the PTC-containing mRNA, thus avoiding the production of toxic mutant proteins. Although the exact mechanism of NAS had not been reported, UPF1 plays an important role in NAS [34]. Together with our findings that UPF1 did not play a significant role in NMTR, all of these findings indicate that (1) NMD, NAS, and NMTR play important roles in the inhibition of deleterious mutant protein production, and (2) unlike NMD and NAS, UPF1 and UPF2 do not a play key role in NMTR, suggesting novel factor(s) or pathways exist in the NMTR.

In conclusion, we demonstrated three different molecular pathways of PTC-containing mRNAs. We propose a novel mechanism of gene expression regulation for PTC-containing mRNAs, in which the deleterious transcripts are regulated either by NMD or NMTR. Future studies of the NMD and NMTR control mechanism will enable us to better understand the reason for specific protein expression among the numerous mRNA isoforms, as well as the selective cellular control mechanism of protein expression.

## Materials and Methods

### Cells and media.

Ten cell lines were obtained from either the American Type Culture Collection (ATCC; http://www.atcc.org) or the Korean Cell Line Bank (KCLB; http://cellbank.snu.ac.kr). Seven cell lines (LS174T, HCT-8, SNU C2A, SNU C4, DLD-1, HCT116, and LOVO) were MMR-deficient, and three (NCI-H508, SW480, and HT29) were MMR-proficient in terms of their MSI status, as determined by previous studies [35,36]. We confirmed the presence of MSI using *BAT26* and *BAT25* markers. Cells were grown in RPMI supplemented with 10% FBS (Life Technologies, Grand Island, New York, United States), penicillin, and streptomycin at 37 °C in 5% CO_2_.

### Identification of MSI and frameshift mutations of target genes.

Genomic DNA and cDNA preparation, analysis of MSI, and identification of target gene frameshift mutations were performed as described previously [37].

### NMD inhibition by puromycin treatment.

We used puromycin (Sigma, St. Louis, Missouri, United States) to inhibit the synthesis of proteins involved in NMD. Cells were grown to 80% confluence and then treated with 30-μg/ml puromycin. Six hours later, the cells were harvested, and total RNA was isolated using an RNeasy Mini kit (QIAGEN, Valencia, California, United States) according to the manufacturer's instructions.

### UPF1 and UPF2 targeting by siRNAs.

Twenty-one–nucleotide RNAs were chemically synthesized using a Silencer siRNA Construction kit (Ambion, Austin, Texas, United States). Synthetic oligonucleotides were deprotected and gel-purified. HCT116 and SNU C2A cells growing in six-well dishes were transfected with 50 nM siRNA and oligofectamine (Invitrogen, Carlsbad, California, United States) according to the manufacturer's protocol. For RT-PCR analysis, total RNA was harvested 48 h after siRNA transfection. Targeted nucleotides, numbered relative to the start codon, were as follows: *rent1/UPF1,* 1,879–1,901 (5′-AAGATGCAGTTCCGCTCCATTTT-3′); *rent2/UPF2,* 1,423–1,445 (5′-AAGGCTTTTGTCCCAGCCATCTT-3′); and *luciferase GL2,* 153–173 (5′-AACACGTACGCGGAATACTTCGA-3′). The inhibition of UPF1 and UPF2 expression by siRNA targeting was evaluated by semi-quantitative RT-PCR or Western blotting.

### Construction of TGFBR2 and MARCKS expression vectors.

To study expression of TGFBR2, we selected the secreted expression vector, pSecTag2B (Invitrogen). The vector was cut by Hind III, and the FLAG oligonucleotide was inserted into the Hind III site to allow for specific immunodetection, thereby creating the pSecTag-FLAG vector. The complete coding sequence of full-length TGFBR2 begins with the first ATG at codon 1 and encodes a 568–amino acid protein, with a stop codon at 569. Mutant TGFBR2 with a 1-bp deletion at ten adenosine repeats results in a premature stop at codon 162 (Figure S1). We constructed wild-type (constructs K and M) and truncated TGFBR2 expression vectors (constructs L, N, O, and P).

Wild-type TGFBR2 cDNA was obtained from a 293T cell line and mutant (1-bp deletion) TGFBR2 cDNA was obtained from a HCT116 cell line by RT-PCR and then cloned using a T&A cloning kit (RBC, Taipei, Taiwan). Construct K (TGFBR2(WT)-cDNA) and construct L (TGFBR2(−1)-cDNA) were generated by inserting the Hind III fragment from the TGFBR2 cDNA into the Hind III site of pSecTag-FLAG.

In order to analyze splicing and subsequent protein expression, we inserted wild-type and mutant *TGFBR2* genomic DNA into the expression vectors. To generate constructs M (TGFBR2(WT)-splicing) and N (TGFBR2(−1)-splicing), exons 1 to 7, except exon 3, of *TGFBR2* were obtained by PCR using 293T cell genomic DNA and cloned into the yT&A cloning vector (RBC). Exon 3 of *TGFBR2* was obtained from LOVO and 293T DNA by PCR, because a 1-bp deletion in the cMNR exists in exon 3 of *TGFBR2* in LOVO. All primers for cloning were designed to contain more than 100 bp of intron sequence on each side of the exon boundary to ensure accurate splicing of the construct. These exon fragments were ligated to each other using a restriction enzyme site in the multiple cloning site (MCS) of the yT&A vector. In the case of exon 1, deletion of the signal peptide was performed using Dpn1 for N-terminal FLAG tagging. The ligated genomic construct of TGFBR2 was inserted into the pSecTag-FLAG vector. To generate construct O (TGFBR2(−1)-irrelevant), exon 4 to exon 7 of TGFBR2(−1)-splicing was deleted by cutting with BstX1, and the remaining construct was self-ligated. To generate construct P (TGFBR2(−1)-irrelevant-F), cDNA from exon 4 to exon 7 of TGFBR2 was inserted into the pSecTag-FLAG vector using EcoR1 and Pst1 restriction enzyme sites, and then genomic DNA from exon 1 to exon 3 was inserted into the same vector using the BamH1 restriction enzyme site.

For the expression study of MARCKS, the expression vector pcDNA3.1(+) (Invitrogen) was cut by Hind III, and the FLAG oligonucleotide was inserted at the Hind III site to allow for specific immunodetection, thereby creating the pcDNA-FLAG vector. In order to analyze splicing and subsequent protein expression, we inserted wild-type and mutant *MARCKS* genomic DNA into expression vectors. To generate constructs P (MARCKS(WT)-splicing) and Q (MARCKS(−2)-splicing), exon 1 of *MARCKS* was obtained through PCR using genomic DNA of 293T and cloned into the yT&A cloning vector (RBC). Exon 2 of *MARCKS* was obtained from SNU C2A by PCR, since 2-bp monoallelic deletions in the cMNR exist in exon 2 of *MARCKS* in SNU C2A. These exon fragments were subcloned into the expression vector pcDNA-FLAG. Primer sequences are shown in Table S3.

### Transfection.

HCT116 and HeLa cells (2 × 10^6^) were transiently transfected using Lipofectamine 2000 (Invitrogen) with the specific construct plasmid and pSecTag-FLAG vector in a 60-mm plate. Cells were harvested 2 d later. Protein was purified from half of the cells using passive lysis buffer (Promega, Madison, Wisconsin, United States), and total RNA was purified from the other half using TRIzol Reagent (Invitrogen).

### Western blotting.

Whole lysates from cell lines were prepared using passive lysis buffer (Promega). Thirty micrograms of the total protein lysates were loaded into each lane, size-fractionated by SDS-PAGE, and then transferred to a PVDF membrane that was blocked with TBST containing 5% skim milk. Primary antibodies against GAPDH (Trevigen, Gaitherburg, Maryland, United States), FLAG (Sigma-Aldrich), hRad50 (Ab13B3; Gene Tex), MARCKS (Santa Cruz Biotechnology, Santa Cruz, California, United States), or hMSH6 and hMSH3 (BD Bioscience, Mountain View, California, United States) were incubated for 1 h at room temperature. After washing, membranes were incubated with HRP-conjugated secondary antibody (Santa Cruz Biotechnology), washed, and then developed with ECL-Plus (Amersham Pharmacia Biotech, Little Chalfont, United Kingdom).

### RPA.

RNA samples were extracted from each cell line using TRIzol Reagent (Invitrogen). RPA was carried out according to the instructions provided by the manufacturer (RiboQuant RPA kit; BD Biosciences) using 20 μg of total RNA per sample. The template antisense ^32^P-labeled RNA probes were specific for *TGFBR2* (unprotected, 271 nt; protected, 254 nt), *hRad50* (371, 353), *hMSH6* (332, 312), and *GAPDH* (134, 120) mRNA. *GAPDH* was used as an internal control. The intensity of specific bands corresponding to individual riboprobes was determined by densitometry. All RPAs were evaluated at different exposures, and only bands that were within the linear range of the film were analyzed.

### Northern blotting.

Total RNA was extracted from HCT116 with TRIZOL Reagent (Invitrogen). Full-length *TGFBR2* cDNA was amplified by RT-PCR using 293T total RNA. Expression of the exogenous *TGFBR2* construct mRNA was analyzed by Northern blot analysis using 10 μg of total RNA according to standard protocols.

### Polysome assay.

Cultures were supplemented with 100-μg/ml cycloheximide for 24 h post-transfection and incubated for 5 min at room temperature. HeLa cells were washed three times with ice-cold PBS containing 100-μg/ml cycloheximide. HeLa cells were collected by scrapping in PBS, transferred to Eppendorf tubes for additional washes, and then lysed in lysis buffer (15 mM Tris-Cl [pH 7.4], 3 mM MgCl_2_, 10 mM NaCl, 0.5% Triton X-100, 100-μg/ml cycloheximide, 1-mg/ml heparin, and 200 U RNasin [Promega]). When indicated, puromycin (100 μg/ml) was added to the cultures 2 h prior to harvesting, and cycloheximide was omitted from the gradient. For each construct, lysates from two 100-mm dishes were pooled into a microcentrifuge tube and incubated for 10 min on ice with occasional mixing. Nuclei and debris were removed by centrifugation at 12,000*g* for 2 min. Then, 1 ml of each cytoplasmic lysate was layered onto an 11-ml 10%–50% sucrose gradient and centrifuged at 4 °C in an SW40 rotor (39,000 rpm) for 2 h. Sixteen fractions were collected from the top with concomitant measurement of absorbance at 254 nm, using a fraction collection system. RNA was extracted with TRIZOL Reagent and analyzed by RT-PCR.

## Supporting Information

Figure S1 A Schematic Diagram Shows Formation of a Premature Termination Codon Resulting from Frameshift Mutation Schematic diagram of the PTC formation derived from frameshift mutation in the MSI-H tumors. These frameshift mutations generate neo-amino acid sequences in the C-terminal part of the proteins and formation of premature termination codon, respectively.
(1.8 MB JPG)

Figure S2 Expression Profile of Premature Termination Codon-Containing mRNAs Analysis of frameshift mutations and mRNA expression profiles of *TGFBR2, hRad50,* and *hMSH6* in seven MMR-deficient and three MMR-proficient colorectal cancer cell lines. PCR primers were designed to contain mononucleotide repeats of each gene. Frameshift mutations are discovered by a change in length of amplified products due to either the insertion or deletion of mononucleotide repeat units of each gene. Fragments of shifted bands (arrowhead) from three target genes are shown in seven MMR-deficient colorectal cancer cell lines by PCR using genomic DNA. In the RT-PCR, *TGFBR2* showed identical mutant transcripts in the seven MMR-deficient colorectal cancer cell lines, whereas a total loss of the *hRad50* and *hMSH6* mutant transcripts was seen in the same seven MMR-deficient colorectal cancer cell lines. All undetected mutant transcripts reappeared when cells were treated with puromycin. A filled triangle (▴) indicates a shifted band from a 1-bp deletion mutation; a filled inverted triangle (▾) indicates a shifted band from a 1-bp insertion mutation.
(1.0 MB JPG)

Figure S3 Detection of Frameshift Mutant *hRad50* and *hMSH6* Transcripts after NMD Inhibition (A) Effects of siRNA treatment on HCT116 and SNU C2A cell lines. HCT116 and SNU C2A cells were transfected with siRNA duplexes directly against *UPF1, UPF2,* and firefly luciferase (negative control). Seventy-two hours after transfection, cells were harvested and RNAs were extracted. Markedly reduced expressions of *UPF1* and *UPF2* were noted by semi-quantitative RT-PCR. *β-actin* was used as a control.
(B) Frameshift mutation profiles of *hRad50* and *hMSH6* were revealed in HCT116 and SNU C2A colorectal cancer cell lines. PCR primers were designed to contain mononucleotide repeats of each gene. Frameshift mutations are discovered by a change in length of amplified products due to either the insertion or deletion of mononucleotide repeat units of each gene. The HCT116 cell line has a heterozygous deletion of an adenine in the (A)_9_ repeats of hRad50 and a heterozygous deletion of a cytosine in the (C)_8_ repeats of hMSH6. The SNU C2A cell line has a heterozygous insertion of an adenine in the (A)_9_ repeats of hRad50 and a homozygous insertion of a cytosine in the (C)_8_ repeats of hMSH6.
(C) Frameshift mutant transcripts of *hRad50* and *hMSH6* were not detected in HCT116 and SNU C2A cell lines. The mutant transcripts appeared when the cell lines were treated with UPF1 and UPF2 siRNA.
(D) Sequence chromatograms of the shifted bands from hRad50 (filled triangle [▴]) and hMSH6 (filled inverted triangle [▾]) show the deletion of one adenine in the hRad50 (A)_9_ repeats, and the insertion of one cytosine in the hMSH6 (C)_8_ repeats, respectively. A filled triangle (▴) indicates a shifted band from a 1-bp deletion of hRad50 mRNA; a filled inverted triangle (▾) indicates a shifted band from a 1-bp insertion of hMSH6 mRNA; Luci, Luciferase.
(1.4 MB JPG)

Figure S4 Western Blotting Analysis of NMD-Escape Proteins from hMSH3 hMSH3 wild-type proteins were detected in the cell lines containing wild-type alleles. However, no truncated proteins of hMSH3 were detected in the cell lines with frameshift mutation. The hMSH3 antibody detected the truncated proteins from the cell lines transfected with mutant hMSH3 cDNA, indicating that antibodies used in our experiments specifically react with the N-terminal region of hMSH3 protein. GAPDH was used as a loading control.
*Mutation status of each gene: (−1) denotes a 1-bp deletion in the cMNR, (w) denotes no mutation in the cMNR, and (+1) denotes a 1-bp insertion in the cMNR. Control denotes the HeLa cell line transfected with mutant hMSH3 cDNA vector.
(562 KB JPG)

Table S1 Frameshift Mutation Status of NMD Target Genes in Colorectal Cancer Cell Lines (53 KB DOC)

Table S2 Primers of the 13 Genes Used for RT-PCR Analysis (38 KB DOC)

Table S3 Primers of the *TGFBR2* and *MARCKS* Used for Cloning (35 KB DOC)

### Accession Numbers

The GenBank (http://www.ncbi.nlm.nih.gov/Genbank) accession numbers for the gene products discussed in this paper are as follows: ABCF1 (NM_001090), ACVR2 (NM_001616), EIF4A3 (NM_014740), GAPDH (NM_002046), hMSH3 (NM_002439), hMSH6 (NM_000179), hRad50 (NM_005732), MARCKS (NM_002356), PRKWNK1 (NM_018979), RFC3 (NM_002915), SEC63 (NM_007214), TAF1B (NM_005680), TCF-4 (NM_030756), TGFBR2 (NM_003242), UPF1 (NM_002911), UPF2 (NM_015542), and Y14 (NM_005105).

## Abbreviations

bp: base pair
cMNR: coding mononucleotide repeats
EJC: exon junction complex
MMR: mismatch repair
MSI: microsatellite instability
MSI-H: high microsatellite instability
NAS: nonsense-mediated altered splicing
NMD: nonsense-mediated mRNA decay
NMTR: nonsense-mediated translational repression
nt: nucleotide
PTC: premature termination codon
RPA: ribonuclease protection assay
RT-PCR: reverse transcriptase PCR
siRNA: small interfering RNA
