# Supplementary material for: Selective Translational Repression of Truncated Proteins from Frameshift Mutation-Derived mRNAs in Tumors
Source: PLoS Biol. 2007 Apr 24;5(5):e109. doi: 10.1371/journal.pbio.0050109 (PMC1854916; doi:10.1371/journal.pbio.0050109)
Supplement: Supplementary file 1 [file pbio.0050109.st001.doc]

**Table S1. Frameshift mutation status of NMD target genes in colorectal cancer cell lines**

| **Gene**  **Cell line** | ***ABCF1*** | ***hMSH6*** | ***hRad50*** | ***PRKWNK1*** | ***RFC3*** | ***SEC63*** | ***hMSH3*** | ***TAF1B*** | ***TGFBR2*** | ***ACVR2*** | ***MARCKS*** | ***TCF-4*** |
| --- | --- | --- | --- | --- | --- | --- | --- | --- | --- | --- | --- | --- |
| **LS174T*** | -1/W | -1/w | +1/w |  | -1/W | -2/W |  | -2/W | -1 | -1/w | -1 | -1/w |
| **HCT-8*** |  |  |  |  | -1 |  |  |  | -1/w | -1/w |  |  |
| **NCI-H508** |  |  |  |  |  |  |  |  |  |  |  |  |
| **SNU C2A*** |  | +1 | -1/w | -1/W |  | -1 |  | -2/W | -1 | -1/w | -2/w | -1 |
| **SNU C4*** | -1/W | -1/w | -1/w | -1/W | -1/W | -1 | -1 | -2/W |  | -1/w | -1 | -1/w |
| **DLD-1*** |  |  |  |  | -1 |  |  |  | -1/w | -1/-2 |  |  |
| **HCT116*** | -2/W | +1/w | -1/w | -1/W | -1/W | -1/-3 | -1 | -2/-3/W | -1 | -1 | -1/w |  |
| **LOVO*** | -1/W |  | -1/w |  | -1/W | -1/W |  | -1/W | -1/-2 | -1 | -1 | -1/w |
| **SW480** |  |  |  |  |  |  |  |  |  |  |  |  |
| **HT29** |  |  |  |  |  |  |  |  |  |  |  |  |

***** MMR-deficient colorectal cancer cell line
