# Supplementary material for: Selective Translational Repression of Truncated Proteins from Frameshift Mutation-Derived mRNAs in Tumors
Source: PLoS Biol. 2007 Apr 24;5(5):e109. doi: 10.1371/journal.pbio.0050109 (PMC1854916; doi:10.1371/journal.pbio.0050109)
Supplement: Supplementary file 2 [file pbio.0050109.st002.doc]

**Table S2.** Primers of the 13 genes used for RT-PCR analysis

| **Gene name** | **Accession No.** | **Forward primer (5’-3’)** | **Reverse primer (5’-3’)** | **PCR product**  **length** |
| --- | --- | --- | --- | --- |
| *ABCF1* | NM_001090 | CAGCAGCAGCAACAGCAA | GCGGGTACTTCATCCTCCTC | 158 |
| *ACVR2* | NM_001616 | GTTGCCATTTGAGGAGGAAA | CCAGCATGTTTCTGCCAATA | 114 |
| *hMSH3* | NM_002439 | CCAGCTATCTTCTGTGCATCTC | CAAACACAACCTCGCCTGT | 114 |
| *hMSH6* | NM_000179 | ATCGCAGTGTTGGATGTTTT | CGTAATGCAAGGATGGCGT | 141 |
| *hRad50* | NM_005732 | AACTGCGACTTGCTCCAGAT | CAATTATGCTTTGCCTCATGG | 111 |
| *MARCKS* | NM_002356 | AAGCGAACGGACAGGAGAAT | CCAGCCTCCTTCTTGTTCTTC | 448 |
| *PRKWNK1* | NM_018979 | GAAATGGTAGAGTCTGGGTATG | GCTGTTTGAGACTGCTCTCTTC | 160 |
| *RFC3* | NM_002915 | TGGTGTTGGAGTGGAAAAAT | CTCGGTCACTATTTCCAGCA | 137 |
| *SEC63* | NM_007214 | GGAGGATGGCAACAGAAGAG | CAGCTTCATTCCCAACGACT | 151 |
| *TAF1B* | NM_005680 | GCACTTCTTGCCACAATGTT | CAATCCCAGCCTTTTTCAGT | 130 |
| *TCF-4* | NM_030756 | GGGACAAGCAGCCGGGAG | CACCTTGTATGTAGCGAACGC | 186 |
| *TGFBR2* | NM_003242 | CAAGCTCCCCTACCATGACT | TGCTGGTGTTATATTCTTCTGA | 158 |
| *GAPDH* | NM_002046 | GCCTCAAGATCATCAGCAAT | TTCAGCTCAGGGATGACCTT | 247 |
