# Supplementary material for: Selective Translational Repression of Truncated Proteins from Frameshift Mutation-Derived mRNAs in Tumors
Source: PLoS Biol. 2007 Apr 24;5(5):e109. doi: 10.1371/journal.pbio.0050109 (PMC1854916; doi:10.1371/journal.pbio.0050109)
Supplement: Supplementary file 3 [file pbio.0050109.st003.doc]

**Table S3.** Primers of the *TGFBR2* and *MARCKS* used for Cloning

| **Gene name** | **Fragment** | **Forward primer (5’-3’)** | **Reverse primer (5’-3’)** |
| --- | --- | --- | --- |
| *TGFBR2* | Exon 1 | CGCTGAGTTGAAGTTGAGTGAG | ACAAAGGGGACCAAACGTG |
|  | Exon 2 | CCCAGCACTCCGTACTATGC | CACTATGGGGTGCTGAGGAG |
|  | Exon 3 | ACGTCTGCTCCAGGTGATGTT | TGTATGCAATCCACCACAGG |
|  | Exon 4 | TCTAGAGCAGGGGATGACGAAC | AATATTCCCTAGACCAGTGTCCAGA |
|  | Exon 5 | CCATAAGTCTTGGAGTTTCAGG | TTGCCCAATAAAACAATGTCA |
|  | Exon 6 | CCCAACCACTCCTTCCTTCT | AAATGTCCTCCGAATGGCTA |
|  | Exon 7 | CATGGGGAATTT CAGGTGT | TCCTGCTGCCTCTGTTCTTT |
|  | cDNA | CCACCGCACGTTCAGAAG | AGCCTGCCCCATAAGAGCTA |
| *MARCKS* | Exon 1 | CGAACTACACTTGGGCTCCT | CCACGCCATTTTAGACGATT |
|  | Exon 2 | CTTGGGGGCTACAGTTGTGT | CAAGGCAGAGGAAGAAGGTG |
